# We’re Lesbian Pioneers- We Can Manifest Good Death: EOL Experiences of Older LGB Women Who Have Lost a Spouse or Partner

**DOI:** 10.1093/geroni/igaa057.2067

**Published:** 2020-12-16

**Authors:** Korijna Valenti, Leah Janssen

**Affiliations:** 1 Miami University, Oxford, Ohio, United States; 2 Miami University, Hamilton, Ohio, United States

## Abstract

Preparing for end-of-life (EOL) and identifying support systems are ways older lesbian, gay, and bisexual women (LGB) women can assert agency over EOL. This paper presents qualitative data from a sample of older LGB women 60+ who have lost a spouse or partner. Thematic analysis revealed four main categories of concern: 1) advance care documents and wills; 2) interactions with healthcare professionals; 3) discussions about EOL; and 4) social network support. The work also analyzed participants’ discussions of how they have changed since losing a loved one and how they now view preparation for EOL. Findings reveal the need for better communication between healthcare professionals and LGB women, better understanding of care options (e.g. hospice, palliative care) and deeper EOL conversations among loved ones. This work critically engages queer gerontology, providing an important foundation of how to better understand how older LGB women perceive needs and preferences for their EOL.

